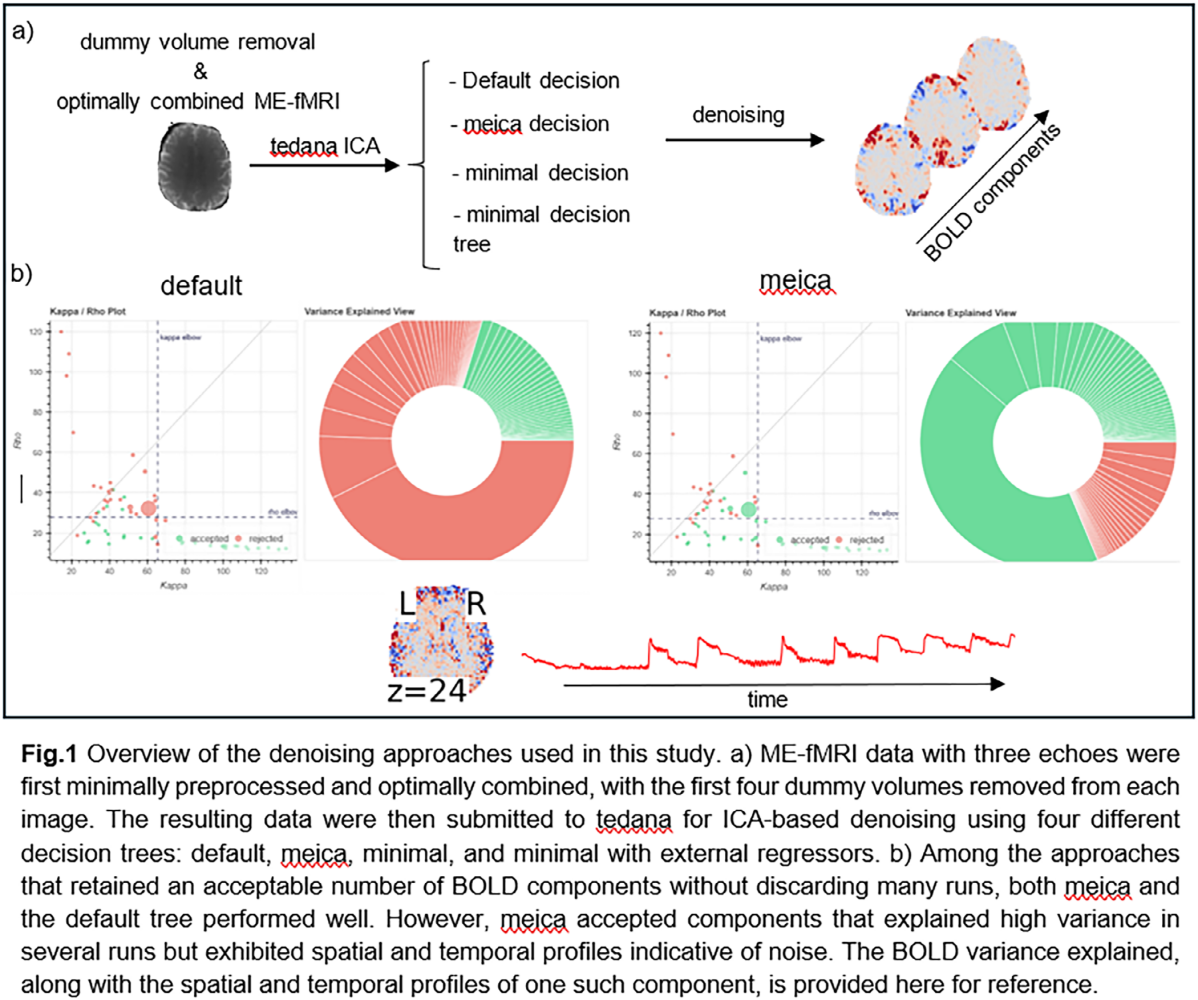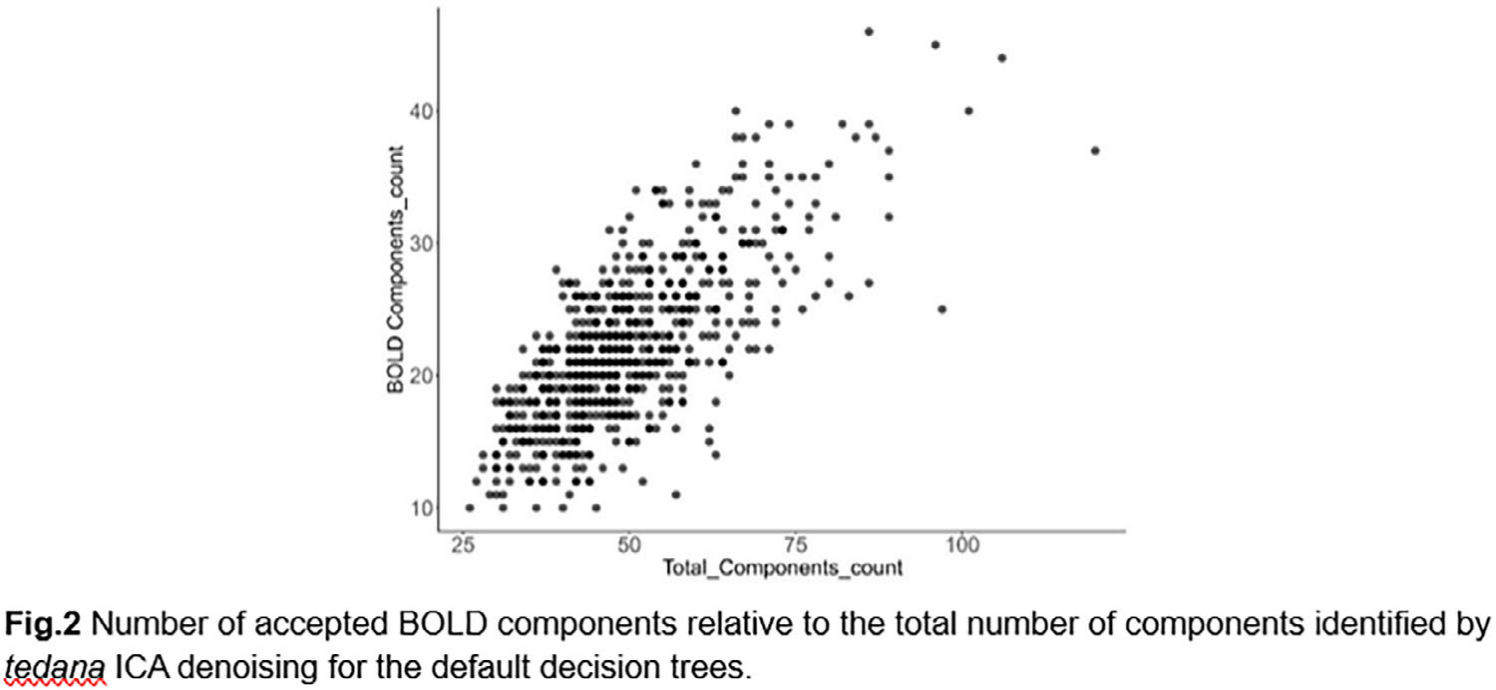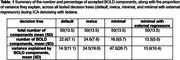# Multi‐echo resting state fMRI processing of the PREVENT‐AD cohort: an open science initiative

**DOI:** 10.1002/alz70861_108898

**Published:** 2025-12-23

**Authors:** Mohammadali Javanray, Alexandre Pastor‐Bernier, Jordana Remz, Jennifer Tremblay‐Mercier, Alexa Pichet Binette, Pierre Bellec, Sylvia Villeneuve, the PREVENT‐AD Research Group

**Affiliations:** ^1^ McGill University, Montreal, QC Canada; ^2^ McGill Integrated Program in Neuroscience (IPN), Montreal, QC Canada; ^3^ Douglas Mental Health University Institute, Montreal, QC Canada; ^4^ Centre for Studies on Prevention of Alzheimer's disease (StoP‐AD Centre), Douglas Mental Health Institute, Montreal, QC Canada; ^5^ Douglas Mental Health University Institute, Centre for Studies on the Prevention of Alzheimer's Disease (StoP‐AD), Montréal, QC Canada; ^6^ Centre de Recherche de l’Institut Universitaire de Gériatrie de Montréal, Montréal, QC Canada; ^7^ Department of Physiology and Pharmacology, Université de Montréal, Montréal, QC Canada; ^8^ Centre de recherche de l'institut universitaire de gériatrie de Montréal (CRIUGM), Montréal, QC Canada; ^9^ Centre de Recherche de l'Institut Universitaire de Gériatrie de Montréal, Montréal, QC Canada; ^10^ Université de Montréal, Montréal, QC Canada; ^11^ Department of Neurology and Neurosurgery, McGill University, Montreal, QC Canada; ^12^ Department of Psychiatry, McGill University, Montreal, QC Canada; ^13^ Centre for Studies on Prevention of Alzheimer's disease (StoP‐AD Centre), Montreal, QC Canada; ^14^ McGill University Research Centre for Studies in Aging, McGill University, Montreal, QC Canada; ^15^ Centre for Studies on Prevention of Alzheimer's Disease (StoP‐AD Centre), Douglas Mental Health University Institute, Montréal, QC Canada

## Abstract

**Background:**

PREVENT‐AD is launching a second wave of open science data sharing, offering neuroimaging derivatives including resting‐state multi‐echo functional MRI (ME‐fMRI) from 348 older adults with a family history of Alzheimer’s disease (AD), who were cognitively healthy at enrolment, and followed for up to 13 years. ME‐fMRI improves the separation of BOLD and non‐BOLD signals, and processing pipelines are being optimized to process these data. To process the PREVENT‐AD ME‐fMRI data for the open science initiative, several strategies were evaluated, and the best‐performing approach was selected.

**Method:**

A total of 666 ME‐fMRI scans from 300 participants were acquired using a 3T SIEMENS MAGNETOM Prisma_fit scanner at the Cerebral Imaging Centre, Douglas Mental Health University Institute (Montreal, Canada). Scans were minimally preprocessed with fMRIPrep. Dummy volumes were removed from both the optimally combined images and each single‐echo image. Data were then denoised using tedana’s independent component analysis (ICA)‐based approach (Figure 1), applying four different decision trees: default, meica, minimal, and minimal with external regressors. The optimal decision tree was selected based on 1) retaining at least 10 BOLD components per run, 2) maximizing variance explained by BOLD components, 3) favorable spatial and temporal profiles of components, and 4) preserving as many runs as possible.

**Result:**

On average, tedana ICA retained 50.0 total number of components (SD = 13.5). The *minimal* and minimal_external_regressors trees led to the exclusion of 54 and 124 runs, respectively, failing the 10‐BOLD component criterion. The meica tree retained the highest number of BOLD components, with an average of 24.6 (SD = 7.6), but accepted components that were rejected by the default tree (Table 1). These additional components exhibited spatial and temporal profiles indicative of noise (Figure 1). The default decision tree yielded the second highest average number of retained BOLD components (22; SD = 7.1) (Figure 2), and did not discard any runs.

**Conclusion:**

The tedana ICA denoising using the default decision tree retained an appropriate number of components while effectively identifying and rejecting noise‐related components and preserving sessions. Therefore, this approach was selected to process the PREVENT‐AD cohort ME‐fMRI data for open science data sharing.